# The lncRNA CRNDE is regulated by E2F6 and sensitizes gastric cancer cells to chemotherapy by inhibiting autophagy

**DOI:** 10.7150/jca.65871

**Published:** 2022-08-08

**Authors:** Feifei Zhang, Qian Chen, Peng Chen, Chaoqun Liu, Hui Wang, Liang Zhao

**Affiliations:** 1 Department of Plastic and Aesthetic Surgery, Nanfang Hospital, Southern Medical University, Guangzhou, 510515, China.; 2 Department of Pathology, Shunde Hospital, Southern Medical University (The First People's Hospital of Shunde), Foshan, China.; 3 Department of Pathology & Guangdong Province Key Laboratory of Molecular Tumor Pathology, School of Basic Medical Sciences, Southern Medical University, Guangzhou, China.; 4 Department of Medical Oncology, Affiliated Tumour Hospital of Guangzhou Medical University, Guangzhou, China.

**Keywords:** LncRNA, CRNDE, Gastric cancer, Autophagy, Chemoresistance

## Abstract

Chemotherapy is an important treatment for gastric cancer (GC), but the primary and secondary drug resistance of tumours to chemotherapy seriously affects its curative effect. In recent years, the relationship between long noncoding RNAs (lncRNAs) and malignant tumours has received increasing attention. Based on accumulating evidence, lncRNAs are involved in the chemoresistance of GC, but the underlying mechanisms remain unclear. In this study, we identified the lncRNA colorectal neoplasia differentially expressed (CRNDE) as an important regulator of autophagy-associated chemoresistance in GC. Mechanistically, overexpression of CRNDE inhibits autophagy and induces apoptosis, thereby sensitizing GC cells to chemotherapy drugs. Moreover, E2F6, a classical transcriptional inhibitor, is confirmed to be upregulated in GC and represses the expression of CRNDE. The E2F6-CRNDE axis is clinically related to chemoresistant GC and poor outcomes in patients with advanced GC. Our findings suggest that the E2F6-CRNDE axis is a viable therapeutic target to protect against chemoresistance in GC.

## Introduction

Gastric cancer (GC) is the fifth most frequently diagnosed cancer in the world and the third leading cause of cancer-related mortality 1, 2. Although surgical resection is the best treatment option for patients with early gastric cancer, more than 60% of patients are diagnosed at advanced stages 3. Chemotherapy plays an important role in the treatment of patients with middle- and late-stage gastric cancer. However, intrinsic or acquired chemoresistance is one of the major obstacles in GC therapy 4. Methods to effectively reverse drug resistance remain a major clinical challenge.

Autophagy is a lysosomal pathway that self-digests proteins and organelles in eukaryotic cells and is a ubiquitous physiological phenomenon 5, 6. According to recent studies, autophagy induced by chemotherapy is a new mechanism of resistance to chemotherapeutic drugs 7. In cancer cells, autophagy provides energy by digesting abnormal proteins and damaged organelles, thereby inhibiting necrosis or apoptosis 8. These prosurvival effects of autophagy protect tumour cells from chemotherapeutic drugs and promote tumour progression.

Long noncoding RNAs (lncRNAs) are a class of RNA transcripts longer than 200 nucleotides 9. Previous studies have revealed that lncRNAs have vital regulatory roles in many aspects of genome function, including alternative splicing and chromosome remodelling 10, as well as diverse pathological processes, including tumour progression 11. In recent years, lncRNAs have emerged as regulators of carcinogenesis, invasion, metastasis and drug resistance in many cancers 12-14.

Colorectal neoplasia differentially expressed (CRNDE) is a long noncoding RNA that is transcribed from chromosome 16 on the strand opposite to the adjacent IRX5 gene 15. CRNDE has been described to be upregulated in various cancers, such as colorectal cancer 15, glioma 16, hepatocellular carcinoma (HCC) 17, lung cancer 18 and gastric cancer (GC) 19. CRNDE plays crucial roles in cancer biological processes, including cell proliferation, migration, invasion and apoptosis 20-24. However, the functions of CRNDE in therapeutical effects on GC and the underlying mechanism remain largely unknown.

In the present study, we identified that CRNDE was downregulated in oxaliplatin (OXA)-resistant and 5-FU-resistant gastric cancer cells. In addition, CRNDE blunted the chemoresistance of gastric cancer cells by promoting apoptosis and inhibiting autophagy flux. Mechanistically, CRNDE was downregulated by E2F6, a generally accepted transcriptional repressor, thereby inducing autophagy and promoting chemoresistance in gastric cancer.

## Materials and Methods

### 2D and 3D culture

The human gastric cancer cell line MGC803, which was purchased from the Cell Bank of the Chinese Academy of Sciences (Shanghai, China), was cultured in RPMI-1640 medium (Gibco, Grand Island, NY, USA) supplemented with 10% foetal bovine serum (PAA Laboratories, Inc., Pasching, Austria). Cells were cultured at 37 °C in an atmosphere containing 5% CO_2_. For 3D cultures, type 1 collagen (PureCol, Advanced BioMatrix) was diluted to 2 mg/ml with RPMI-1640 medium containing 10% FBS. Collagen was divided into 3 layers and spread in 12-well plates. Each layer was 400 μl, and the middle layer contained 5000 cells/ml. Next, 1 ml of medium containing 5 μg/ml oxaliplatin and 200 μg/ml 5-FU was added to each collagen sandwich, and the medium was changed every 2-3 days. When the clones grew for approximately 25 days, a single clone was removed, digested with trypsin, and then transferred to a 6-well plate for further culture. After the cells reached confluence, the cells were digested and repeatedly cultured using the aforementioned 3D method for approximately 6 months.

### Overexpression and RNA interference

Plasmids expressing CRNDE, shCRNDE and siE2F6 were purchased from GenePharma (Suzhou, China). Cells were seeded at a density of 6×10^3^ cells/well in a 6-well plate and cultured until reaching 70% confluence. Afterwards, cells were transfected with 1 mg of siRNA or 4 μg of cDNA using Lipofectamine 2000 reagent for 24 h (Invitrogen; Carlsbad, CA, USA) according to the manufacturer's protocol.

### Stable expression of mRFP-GFP-LC3

The lentivirus vector containing the LV-mRFP-GFP-LC3 reporter was purchased from GenePharma (GenePharma, Suzhou, China). Cells stably expressing mRFP-GFP-LC3 were selected by treatment with puromycin (1 μg/ml). After different treatments, the cells were fixed and then analysed using fluorescence microscopy (LSM880, Carl Zeiss, Jena, Germany).

### Western blot assay

Cells were washed with PBS and isolated with RIPA cell lysis buffer supplemented with protease inhibitors. The protein content was determined using a BCA Protein Quantification Kit (KeyGen Biotech, Nanjing, China). The protein lysate was separated using 10% sodium dodecyl sulfate/polyacrylamide gel electrophoresis and transferred to a PVDF ImmobilonP membrane (Roche). After blocking with 5% skim milk, the membranes were incubated with specific primary antibodies and corresponding secondary antibodies. The intensity of the protein bands was determined using a Gel-pro analyser. Protein levels were quantified using densitometry and analysed with ImageJ software. Antibodies against LC3B (NB100-2220; 1:1000; Novus Biologicals, CO, USA) and GAPDH (60004-1-Ig; 1:1000; Proteintech, IL, USA) were used.

### Real-time PCR (RT-PCR)

Total RNA was extracted from exponentially growing cells using TRIzol® reagent (Invitrogen; Thermo Fisher Scientific, Inc.). Reverse transcription of RNA to cDNAs was performed with PrimerScript^TM^ RT Master Mix (RR036A, Takara, Dalian, China), and RT-PCR was performed using TB Green^®^ Premix Ex Taq^TM^ (RR420A, Takara, Dalian, China) according to the manufacturer's instructions. The relative expression of genes was calculated using the ∆∆CT method.

### Tumour tissue samples

The paraffin-embedded tissue of gastric adenocarcinoma was obtained from Nanfang Hospital, Southern Medical University (Guangzhou, Guangdong Province, China). The medical records of the patients were reviewed to collect the following clinicopathological information: age, sex, pathological stage, and tumour node metastasis (TNM) stage. This study was approved by the Ethics Committee of Southern Medical University, and all aspects of the study followed the guidelines of the Declaration of Helsinki.

### Luciferase reporter assay

Lipofectamine 3000 transfection reagent (Life Technologies, Carlsbad, USA) was used to transfect luciferase reporter vectors into 293T and MGC803 cells plated in 24-well plates. Culture supernatants were harvested for luciferase assays using a luciferase assay kit (Promega, Madison, Wisconsin, USA), and the luciferase activity was measured with a PerkinElmer 2030 Multilabel Reader (PerkinElmer, Waltham, Massachusetts, USA).

### Transmission electron microscopy (TEM)

Cells were fixed for 2 h with 2.5% glutaraldehyde containing 0.1 mol/l sodium cacodylate at 4 °C, incubated with 1% osmium tetroxide, and dehydrated in graded ethanol and acetone solutions. Samples were then embedded, cut into 50-nm sections, and stained with 3% uranyl acetate and lead citrate. Images were obtained using a JEM-1200 TEM (JEOL, Tokyo, Japan).

### CCK 8 assay

Cells (6×10^4^ cells/ml) were seeded on a 96-well plate and incubated with various concentrations of OXA (0, 5, 10, 20, 30, 40, or 50 μg/ml) and 5-FU (0, 50, 100, 250 or 500 μg/ml). After an incubation at 37 °C for 24 h, the cells were analysed using Cell Counting Kit-8 (CCK-8, Dojindo, Japan) and the absorbance (450 nm) was measured according to the manufacturer's protocol.

### Chromatin immunoprecipitation

Chromatin immunoprecipitation (ChIP) assays were performed using a Pierce^TM^ Agarose ChIP Kit (Thermo Scientific, Waltham, MA, USA; catalogue no. 26156) with an anti-E2F6 antibody (rabbit polyclonal, CST, USA; catalogue no. ab53061). The negative control group was normal mouse immunoglobulin G (IgG). The binding sites for E2F6 in the CRNDE promoter were detected using PCR with specific primers.

### Immunohistochemistry (IHC)

IHC was performed to investigate the expression of proteins in human gastric cancer tissues. Sections were incubated overnight with primary antibodies against E2F6 (NBP2-14936, 1:500; Novus Biologicals, CO, USA) at 4 °C. Mayer's haematoxylin was used for nuclear counterstaining. In this study, these slides were reviewed by two or three blinded pathologists.

The immune-stained slides were evaluated and scored as a sum of the staining intensity and percentage of positive tumour cells. Briefly, percentage of positive cells staining was scored as follows: 0%(absent), 1-5%(sporadic), 6-25%(local), 26-50%(occasional), 51-75%(majority) and 76-100% (large majority). The staining intensity of cancer cells was scored as 0 (no staining), 1 (weak staining, light yellow), 2 (moderate staining, yellowish brown), and 3 (strong staining, brown). An intensity score of ≥2 with more than 50% of E2F6-positive cells was used to classify tumours with high expression (or overexpression), whereas <2 in intensity score or <50% of cells with detectable E2F6 expression classified tumours with low expression. The discrepancies (<5%) were resolved by simultaneous re-evaluation.

### *In situ* Hybridization (ISH)

*In situ* hybridization was performed using an ISH kit from Boster (Wuhan, China). Xylene, ethanol and protease were used to immobilize and permeate cells in clinical samples (10 μm) to allow access to biotin-labelled probes. The slides were treated with 30% H2O2 and ddH2O at a ratio of 1:10 for 5 minutes, and then the nucleic acid fragments were exposed to pepsin diluted with 3% citric acid for 20 seconds. The second fixation step was followed by an incubation with 1% paraformaldehyde/0.1 M PBS. Next, the slides were incubated with prehybridization solution at 40 °C for 2 h and then incubated with the lncRNA target probe overnight at 30 °C, followed by two washes with 2× saline sodium citrate. After blocking, biotin-labelled anti-digoxin was added and incubated for 60 minutes. Finally, the slides were stained with DAB, dehydrated with 100% ethanol and xylene, and mounted with xylene-based mounting medium. The slides were recorded with a Pannoramic SCAN instrument (3dhistech, Budapest, Hungary) and analysed using Pannoramic Viewer (3dhistech, Budapest, Hungary).

### Statistical analysis

Data were analysed using SPSS statistical software version 20.0 (IBM Corporation, Armonk, NY, USA). The differences between groups were assessed using Student's* t*-test or one-way ANOVA. Survival data were analysed using the Kaplan-Meier method and log-rank test. Statistical significance was established at P < 0.05.

## Results

### CRNDE is downregulated in chemoresistant GC cells in both 2D and 3D culture

**Figure 1:**
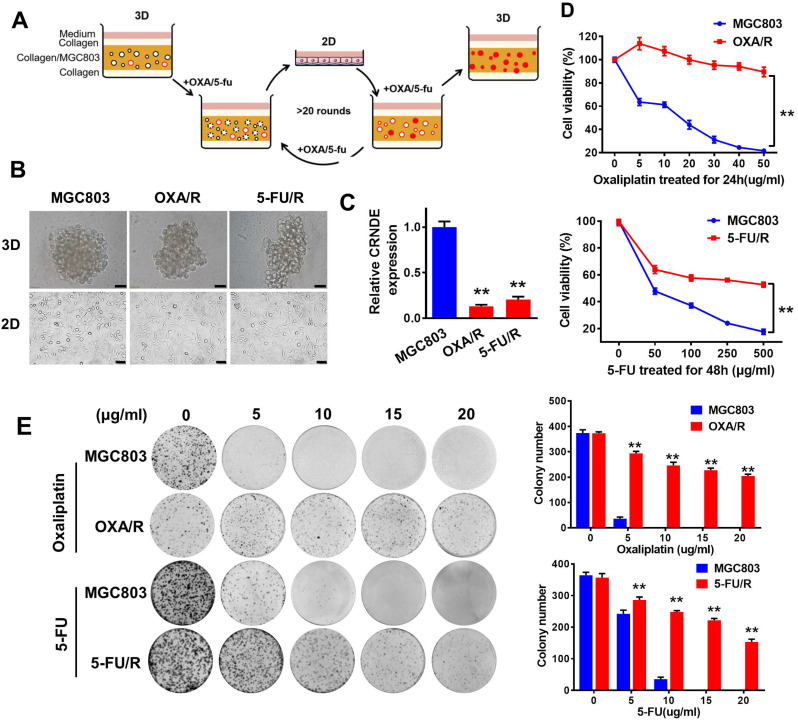
** CRNDE is downregulated in chemoresistant GC cells both in 2D and 3D culture. (A)** A schematic diagram of the experimental method for the establishment of oxaliplatin and 5-FU drug-resistant cell lines in 3D. In 3D type-I collagen culture, more than 95% of GC colonies died in the presence of oxaliplatin (5 µg/ml) or 5-FU (200 µg/ml). In the presence of oxaliplatin and 5-FU, the residual colonies were isolated and passaged repeatedly in 2D and 3D for about 6 months. These clones are named OXA/R or 5-FU/R. **(B)** Sixteen-day old MGC803, OXA/R and 5-FU/R cells were treated with oxaliplatin (5 µg/ml) or 5-FU (200 µg/ml) for 3 days. Representative images from three independent experiments are given. Scale bars, 50 µm. **(C)** RT-PCR analysis of the expression level of CRNDE in OXA/R, 5-FU/R and their parent cells MGC803. **(D)** Cell viability of MGC803, OXA/R and 5-FU/R cells after oxaliplatin and 5-FU treatment were determined by CCK8 assays. **(E)** Representative images of colony formation analysis in MGC803, OXA/R and 5-FU/R cells treated with different concentrations of oxaliplatin and 5-FU for 14 days. The histogram shows the quantification of colony formation test data. **P < 0.01.

By subjecting MGC803 cells to 3D culture in type I collagen with periodic additions of oxaliplatin and 5-FU, we obtained oxaliplatin (OXA/R)- and 5-FU (5-FU/R)-resistant MGC803 cells (Fig. 1A). Our data showed that the two resistant cell lines also exhibited cross-resistance to other chemotherapeutic drugs, such as paclitaxel (TAX), epirubicin, and VP-16 (etoposide) (Fig. S1). In 3D culture, no difference in morphology was observed between OXA/R, 5-FU/R and parental MGC803 cells. However, in 2D culture, OXA/R and 5-FU/R cells showed longer extensions than their parental MGC803 cells (Fig. 1B). RT-PCR results showed lower expression of CRNDE in OXA/R and 5-FU/R cells than in MGC803 cells (Fig. 1C). As expected, OXA/R and 5-FU/R cells remained refractory to oxaliplatin and 5-FU (Fig. 1D&E).

### GC cells with blunted chemosensitivity exhibit increased autophagy

**Figure 2:**
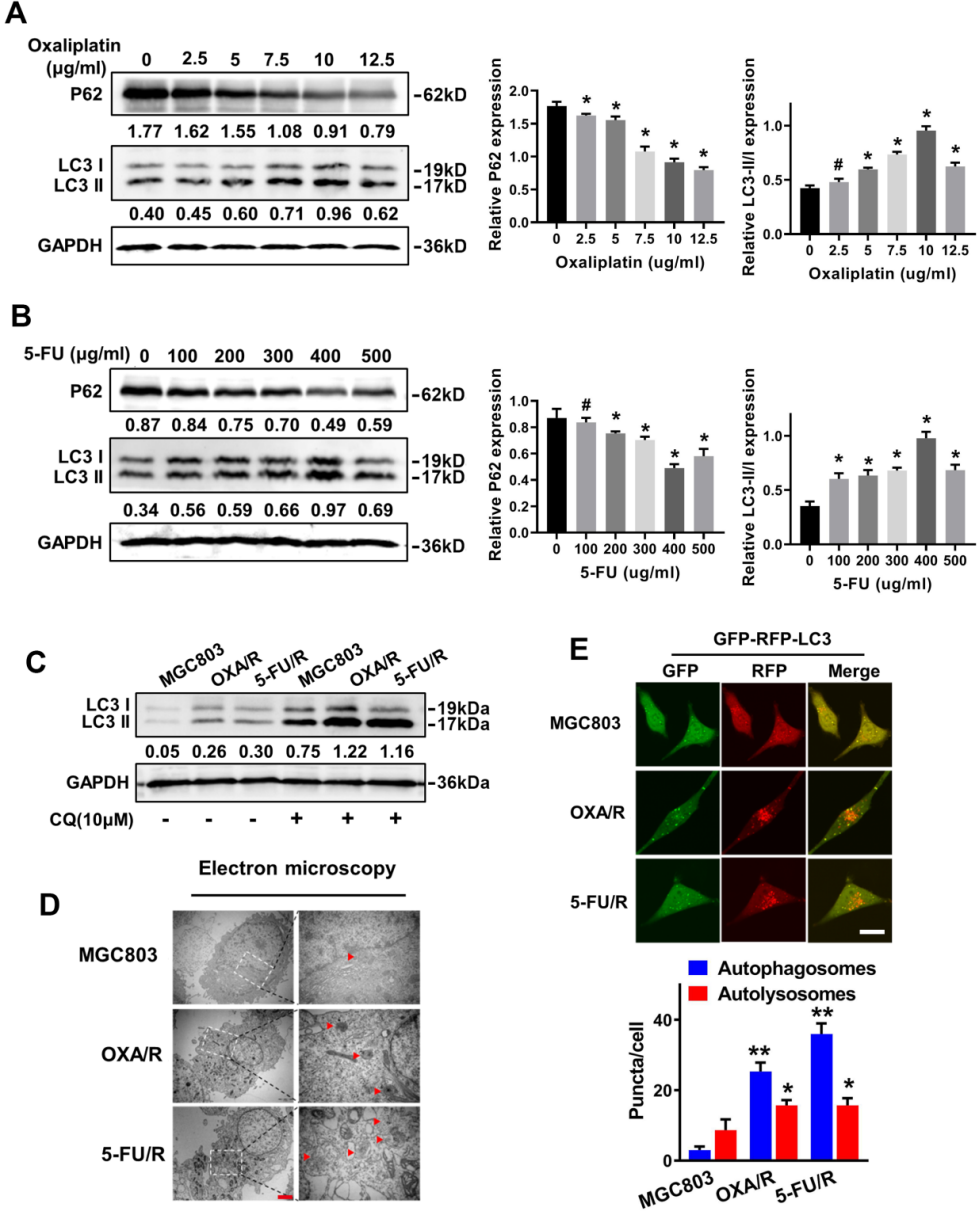
** GC cells with blunted chemosensitivity exhibit increased autophagy. (A, B)** The protein levels of LC3 and p62 in MGC803 cells treated with different concentrations of oxaliplatin and 5-FU were determined by western blot assay. **(C)** The protein levels of LC3 in MGC803, OXA/R and 5-FU/R cells with or without treatment of CQ (10 µM) were determined by western blot assay. The relative protein abundance is conducted through normalizing to GAPDH. **(D)** Autophagy was evaluated in MGC803, OXA/R and 5-FU/R cells using transmission electron microscopy. Scale bars, 2 µm. **(E)** MGC803, OXA/R and 5-FU/R cells that stably expressed mRFP-GFP-LC3 fusion protein were observed by confocal microscopic. Scale bars, 10 µm. *P < 0.05, **P < 0.01, # P > 0.05.

MGC803 cells were treated with different concentrations of OXA and 5-FU for 24 h, and western blot analysis was utilized to detect the expression of the autophagy-related specific markers LC3 and P62 and to reveal the molecular mechanisms contributing to the drug resistance of GC cells. The western blot results showed significantly increased expression of LC3-II and decreased expression of P62 in MGC803 cells treated with 10 µg/ml oxaliplatin and 400 μg/ml 5-FU (Fig. 2A&B). Next, we evaluated the autophagy levels of OXA/R and 5-FU/R cells under basal conditions. As expected, OXA/R cells and 5-FU/R cells exhibited a higher LC3-II to LC3-I ratio than MGC803 cells. After treatment with chloroquine (CQ), an autophagy-lysosomal inhibitor, a further increase in the LC3-II level was observed, especially in OXA/R and 5-FU/R cells, suggesting an increase in the autophagy flux of chemoresistant cells (Fig. 2C). Moreover, transmission electron microscopy (TEM) revealed that the number of autophagic vesicles was markedly increased in OXA/R and 5-FU/R cells compared with parental cells (Fig. 2D). We established gastric cancer cells stably expressing mRFP-GFP-LC3 using a lentiviral vector to localize and assess increased autophagy flux in drug-resistant cells. Consistent with the results described above, we observed an increased number of autophagosomes and autolysosomes in OXA/R and 5-FU/R cells using a fluorescence microscope (Fig. 2E). Collectively, these data supported the hypothesis that the drug-resistant property of gastric cancer cells is related to their enhanced autophagy activity.

### CRNDE impairs autophagy and chemotherapy resistance in OXA/R and 5-FU/R cells

**Figure 3:**
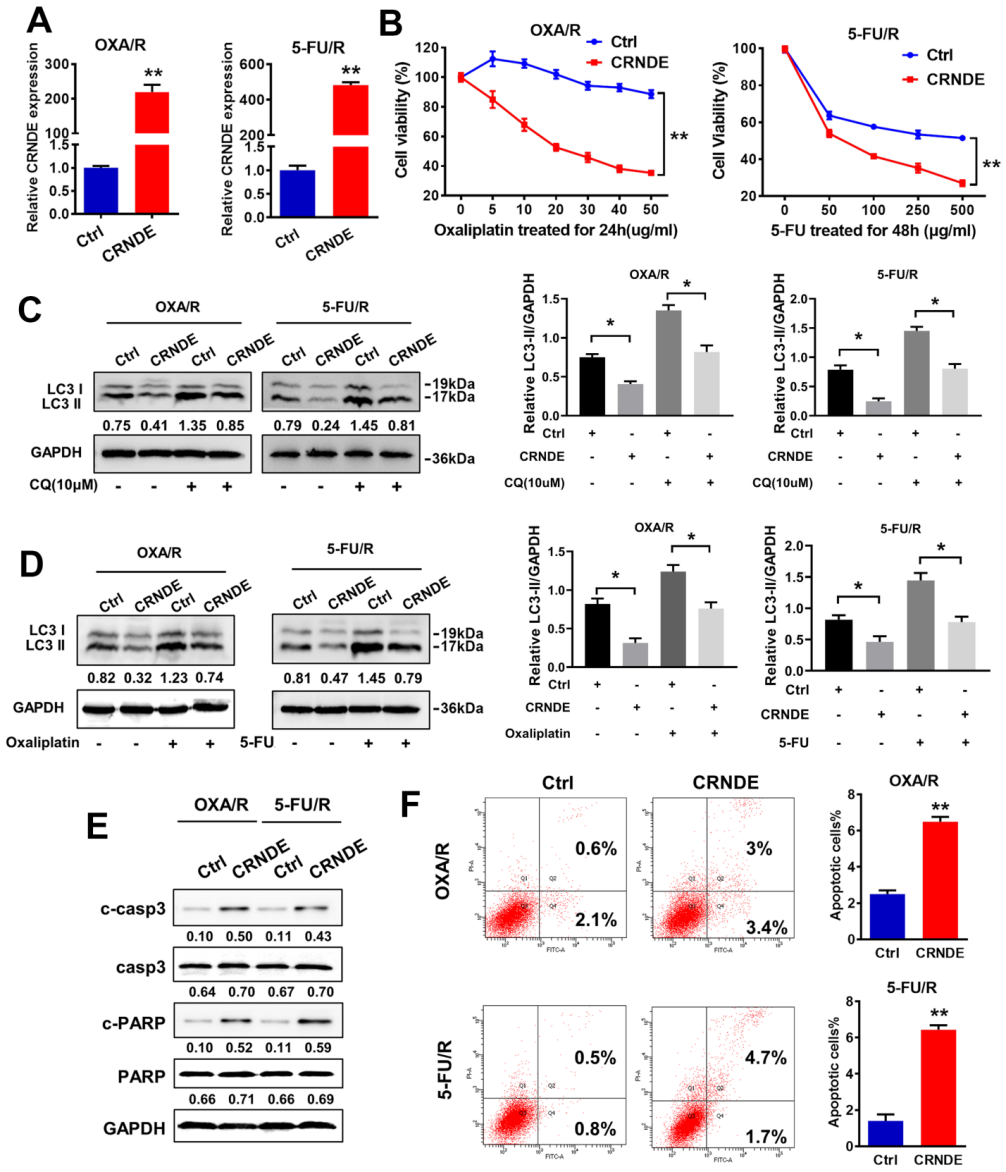
** CRNDE impairs autophagy and chemotherapy resistance in OXA/R and 5-FU/R cells. (A)** RT-PCR analysis of CRNDE expression in OXA/R, 5-FU/R transfected with CRNDE plasmid. **(B)** Cell viability of OXA/R, 5-FU/R transfected with CRNDE plasmid or a control were determined by CCK8 assays. **(C)** The protein levels of LC3 in OXA/R, 5-FU/R transfected with CRNDE plasmid or a control in the presence of CQ were determined by western blot assay. **(D)** The protein levels of LC3 in OXA/R, 5-FU/R transfected with CRNDE plasmid in the presence of 10 µg/ml oxaliplatin or 400 µg/ml 5-FU were determined by western blot assay. The relative protein abundance is conducted through normalizing to GAPDH. **(E)** The protein levels of cleaved-caspase3 and cleaved-PARP in OXA/R and 5-FU/R cells under different transfection were determined by western blot assay. **(F)** Flow cytometric analysis of apoptosis in OXA/R and 5-FU/R cells under different transfection. *P < 0.05, **P < 0.01.

CRNDE and shCRNDE plasmids were used for CRNDE overexpression and CRNDE interference, respectively, to investigate the potential role of CRNDE in the chemoresistance of gastric cancer. We transfected OXA/R and 5-FU/R cells with the CRNDE plasmid, and the transfection efficiency was validated by RT-PCR (Fig. 3A). A CCK-8 drug sensitivity assay was performed and showed that CRNDE sensitized OXA/R and 5-FU/R cells to oxaliplatin and 5-FU (Fig. 3B). Then, we explored whether CRNDE regulate the chemosensitivity of gastric cancer by inhibiting autophagy. The western blot results showed that increased levels of LC3-II following treatment with CQ, while the combination of CRNDE and CQ decreased the expression of LC3-II in OXA/R and 5-FU/R cells (Fig. 3C). Furthermore, treatment with OXA (10 μg/ml) and 5-FU (400 μg/ml) significantly increased the level of LC3-II and triggered autophagy, while CRNDE blunted autophagy compared to cells transfected with a control vector (Fig. 3D). Moreover, knockdown of CRNDE substantially inhibited apoptosis, as indicated by the decreased expression levels of cleaved caspase3 and cleaved PARP in MGC803 cells (Fig. S2A&B). The opposite phenomenon was observed in CRNDE-overexpressing OXA/R and 5-FU/R cells (Fig. 3E). Consistently, the flow cytometry analysis supported the finding that CRNDE stimulates apoptosis in OXA/R and 5-FU/R cells, while knockdown of CRNDE reduced apoptosis in MGC803 cells (Fig. 3F and S2C).

### CRNDE inhibits autophagy-associated chemoresistance in GC cells

**Figure 4:**
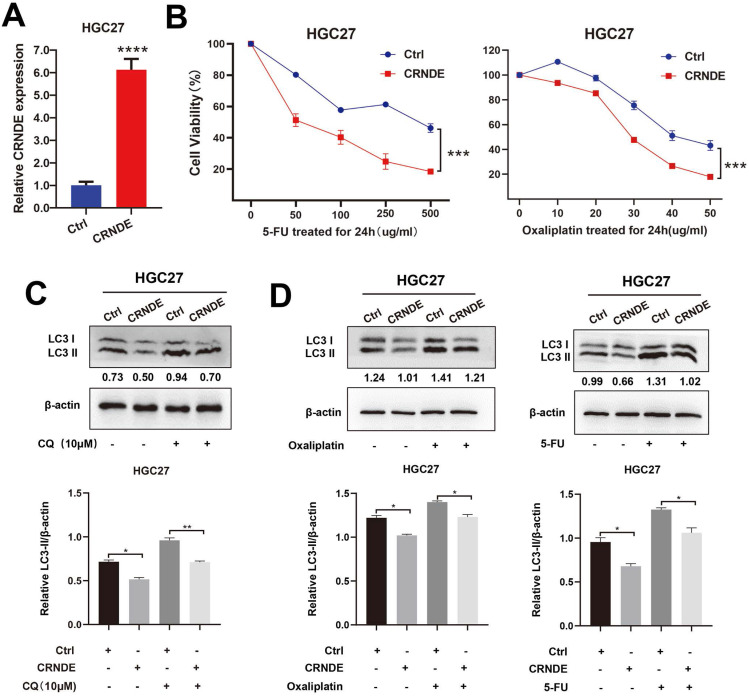
** CRNDE inhibits autophagy-associated chemoresistance in GC cells. (A)** RT-PCR analysis of CRNDE expression in HGC27 cells transfected with CRNDE plasmid. **(B)** Cell viability of HGC27 cells transfected with CRNDE plasmid or a control were determined by CCK8 assays. **(C)** The protein levels of LC3 in HGC27 cells transfected with CRNDE plasmid or a control in the presence of CQ were determined by western blot assay. **(D)** The protein levels of LC3 in HGC27 cells transfected with CRNDE plasmid in the presence of 10 µg/ml oxaliplatin or 400 µg/ml 5-FU were determined by western blot assay. The relative protein abundance is conducted through normalizing to β-actin. *P < 0.05, **P < 0.01, ***P < 0.001, ****P < 0.0001.

The exogenous CRNDE plasmid was also transfected into HGC27 cells (Fig. 4A). CCK-8 drug sensitivity assays demonstrated that CRNDE significantly sensitized the HGC27 cells to 5-Fu and OXA treatment (Fig. 4B). As expected, LC3-II levels induced by CQ were demonstrated to be lower in CRNDE overexpressed HGC27 cells (Fig. 4C). After being treated with chemotherapy drug, higher expression of LC3-II was found in control cells, indicating that CRNDE inhibited autophagy in gastric cancer cell (Fig. 4D).

### E2F6, an upstream transcription factor, regulates CRNDE transcription and inhibits its expression

**Figure 5:**
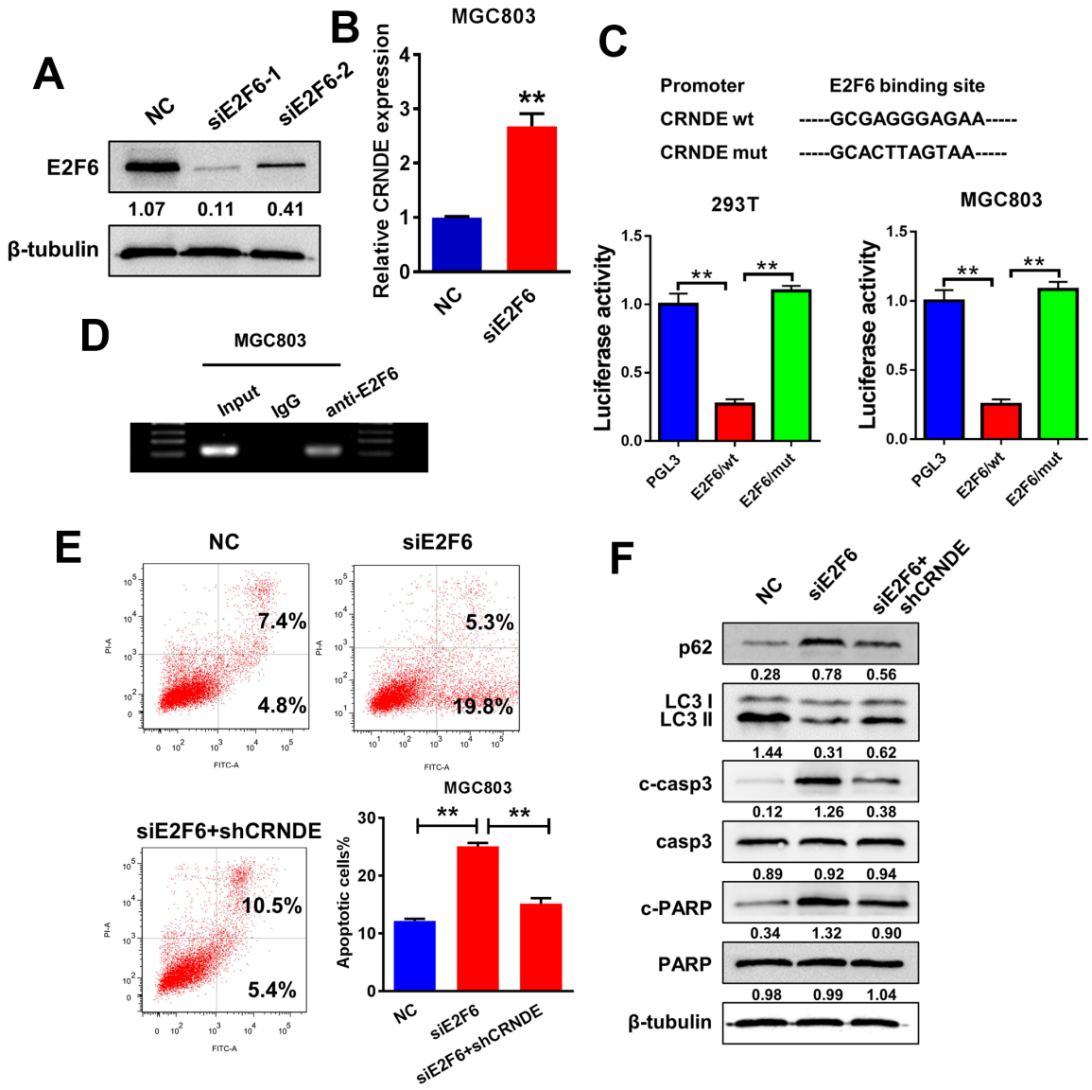
** E2F6, as an upstream transcription factor, regulates CRNDE transcription and inhibits its expression. (A)** Western blot analysis to confirm the efficacy of interference of E2F6. **(B)** RT-PCR analysis of E2F6 expression in MGC803 cells transfected with siE2F6 plasmid. **(C)** Luciferase reporter assays were performed in 293T and MGC803 cells transfected CRNDE wild type or mutants with E2F6. **(D)** ChIP assays were performed to confirm that E2F6 could bind to the promoter of CRNDE. **(E)** Flow cytometric analysis of apoptosis in MGC803 cells under different transfection. **(F)** The protein levels of LC3, p62, cleaved-caspase3 and cleaved-PARP in MGC803 under different transfection were determined by western blot assay. *P < 0.05, **P < 0.01.

The JASPAR database (http://jaspar.genereg.net) was utilized to analyse a 2-kb region upstream of the CRNDE transcription start site and to identify the transcriptional regulatory mechanism of CRNDE expression. Within the putative CRNDE promoter region, E2F6 binding motifs at nucleotides 660-670 were identified. We transfected two individual siRNAs against E2F6 into MGC803 cells to elucidate the role of E2F6 in the regulation of CRNDE (Fig. 5A). Then, we selected si-E2F6-1 for the subsequent experiments due to its higher interference efficiency. The RT-PCR analysis showed that E2F6 knockdown increased the level of CRNDE (Fig. 5B). To further validated that E2F6 regulates the expression of CRNDE, we found that the luciferase activity of the wild-type CRNDE promoter decreased after E2F6 expression was upregulated in 293T and MGC803 cells (Fig. 5C). Furthermore, DNA from immunoprecipitated chromatin showed a significant enrichment in the predicted region compared with the decreased levels in the negative control (IgG) group (Fig. 5D). Then, we examined the effects of E2F6 on CRNDE-mediated chemoresistance. Knockdown of E2F6 induced apoptosis and inhibited autophagy in MGC803 cells, changes that were alleviated by silencing CRNDE expression (Fig. 5E&F). Based on our data, E2F6 inhibits the expression of CRNDE by binding to the CRNDE promoter, thereby promoting autophagy and inhibiting apoptosis.

### The E2F6-CRNDE axis is clinically related to chemoresistant GC and poor outcomes in patients with advanced GC

**Figure 6:**
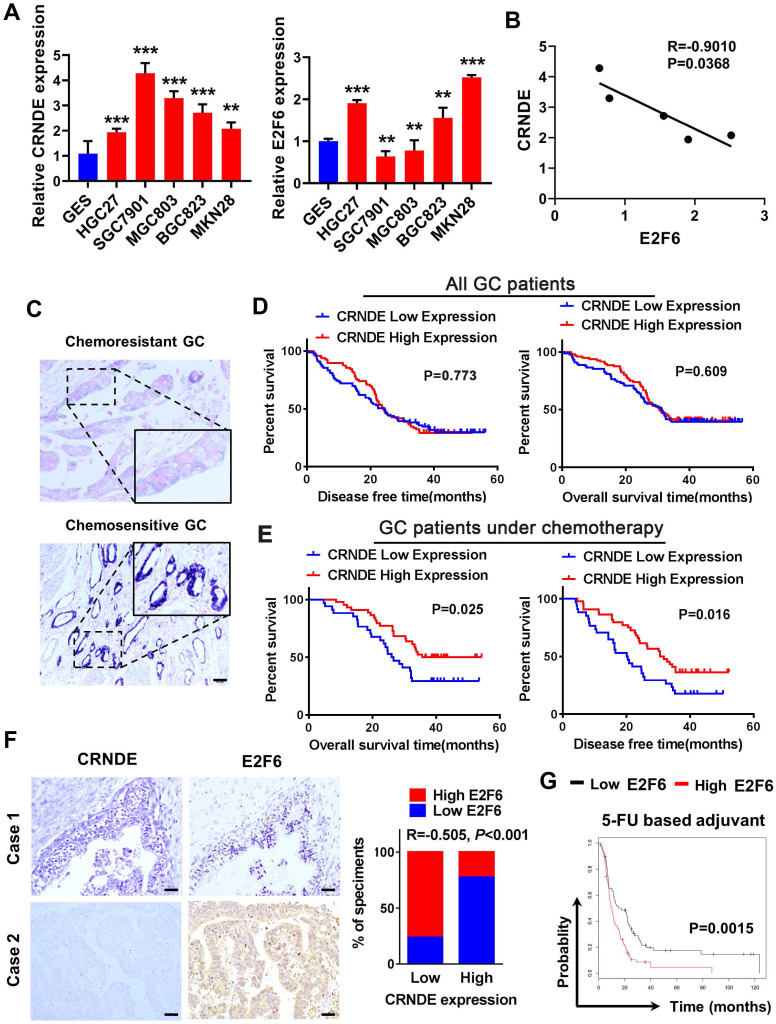
** The E2F6-CRNDE axis is clinically related to chemoresistant GC and poor outcome in advanced GC patients. (A)** The expression of CRNDE and E2F6 were detected by qPCR assay in GC cell lines. **(B)** The correlation of CRNDE and E2F6 was analyzed in GC cell lines. **(C)** The subcellular localization and intensity of CRNDE were examined with *in situ* hybridization (ISH) in sections from GC patients. Scale bar: 50 µm. **(D)** Retrospective analysis of Kaplan-Meier plots for CRNDE expression and the association with disease-free survival (DSF) and overall survival (OS) in GC patients. **(E)** Retrospective analysis of Kaplan-Meier plots for CRNDE expression and the association with disease-free survival (DSF) and overall survival (OS) in GC patients treated with oxaliplatin and 5-FU-based chemotherapy after surgery. **(F)** Representative ISH and IHC staining of GC tissues indicated a negative correlation between CRNDE and E2F6. Bars blow indicates the percentage of patients. Spearman's correlation analysis **(G)** Kaplan-Meier analysis of GC overall survival (OS) corresponding to the expression of E2F6 analysed by the online Kaplan Meier Plotter database (http://kmplot.com/analysis/). *P < 0.05, **P < 0.01, ***P < 0.001.

We detected CRNDE and E2F6 expression in GC cell lines using qPCR, and analyzed their correlation. The results showed that the expression of CRNDE was higher in GC cells than human gastric mucosal epithelial cell line GES (Fig. 6A) and negatively correlated with the expression of E2F6 (Fig. 6B). We examined CRNDE expression in 165 archived paraffin-embedded GC tissues using *in situ* hybridization (ISH). CRNDE expression was detected in both the cytoplasm and nucleus of chemoresistant and chemosensitive GC tissues and it was significantly upregulated in chemosensitive GC tissues compared to chemoresistant GC tissues (Fig. 6C). The Kaplan-Meier survival analysis showed that CRNDE expression was not associated with DFS (disease-free survival) or OS (overall survival) in patients with GC (Fig. 6D). The prognosis of 78 patients with gastric cancer treated with oxaliplatin and 5-FU-based chemotherapy after surgery was analysed. Patients with high CRNDE expression had longer disease-free survival and overall survival (Fig. 6E). IHC and ISH results obtained from 74 archived paraffin-embedded GC tissues indicated that the level of CRNDE was negatively correlated with the level of the E2F6 (Fig. 6F). Finally, we analysed the relationship between E2F6 expression and the prognosis of patients with gastric cancer using the bioinformatics database KM Plotter. The prognosis of patients with high E2F6 expression was significantly worse than that of patients with low expression who were treated with 5-FU and other chemotherapeutic drug-based chemotherapy regimens (Fig. 6G). This finding is consistent with the previous analysis of the relationship between CRNDE and the prognosis of patients undergoing chemotherapy, further confirming the negative correlation between CRNDE and E2F6 expression.

## Discussion

In the present study, we constructed oxaliplatin- and 5-FU-resistant gastric cancer cell lines by establishing a 3D culture model. Compared with the traditional 2D model, the 3D culture model better reflects the spatial structure of tumour cells and the interaction with the extracellular matrix 25. Western blot assays and transmission electron microscopy revealed that OXA/R and 5-FU/R cells presented increased autophagy activity. Autophagy is a catabolic process that degrades and clears damaged organelles, lipids and proteins through the lysosomal pathway 25. Studies have reported that autophagy plays an important role in the process of chemotherapy resistance. On the one hand, cancer cells can recover resources through autophagy to provide raw materials for cell survival and regeneration. On the other hand, cancer cells can achieve self-purification through autophagy and maintain the stability of the intracellular environment and metabolic balance to resist the effect of chemotherapy 26. In addition, accumulating evidence indicates that many chemotherapeutic drugs can induce autophagy in cancer cells 27-29.

As shown in our study, CRNDE, a differentially expressed lncRNA that was first detected in colorectal cancer, was downregulated in OXA/R and 5-FU/R cells. Current reports have shown that CRNDE plays an oncogenic role in many caners. Studies have found that CRNDE is highly expressed in GC, and the survival time of patients with high expression of CRNDE is shorter. Interference with CRNDE expression can inhibit the proliferation and metastasis of GC cells 19, 30. However, in this study, we detected the expression of CRNDE in 165 paraffin-embedded tissue samples of GC patients and found that the expression of CRNDE was not related to the OS of patients. Another study showed that the expression of CRNDE in GC was not different between gastric cancer and normal tissues both in the TCGA database or GEO database, and it was not related to the OS of patients with GC 31, which is consistent with our results. Although CRNDE has been shown to promote chemoresistance in colorectal cancer and hepatocellular carcinoma 32, 33, the regulatory role of CRNDE in GC resistance remains unclear. Interestingly, among patients with GC receiving 5-FU and oxaliplatin chemotherapy, we found that patients with high CRNDE expression experienced longer DFS and OS. Loss- and gain-of-function assays confirmed that CRNDE increased the sensitivity of gastric cancer cells to chemotherapy drugs via a mechanism involving autophagy and apoptosis.

We analysed the UCSC genomics database and Jaspar website to predict potential E2F6 binding sites in the CRNDE promoter and to explore the mechanism of CRNDE downregulation in the drug resistance of gastric cancer. In mammals, nine known members of the E2F transcription factor family have been identified and are involved in cell proliferation, differentiation and apoptosis 34. Among them, E2F6 is recognized as a classical transcriptional inhibitor that exerts its biological function by inhibiting the transcription of downstream genes. In gastric adenocarcinoma, E2F6 is a downstream target gene of miR-31, which is involved in the sensitization of 5-FU induced by high expression of miR-31 35. In prostate cancer, the expression of E2F6 is decreased under the action of moxiflostat. Overexpression of E2F6 can inhibit the apoptosis induced by moxiflostat, while interference with the expression of E2F6 can promote the apoptosis induced by moxiflostat 36. All of the above suggest that E2F6 is a drug resistance related gene. Therefore, we speculate that the low expression of CRNDE may be related to E2F6, and the regulatory relationship between E2F6 and CRNDE is worthy of further study. Mechanistically, the results of the ChIP experiment and luciferase reporter assay confirmed that the CRNDE promoter was occupied by the transcriptional repressor E2F6. The expression of CRNDE increased after interfering with the expression of E2F6, suggesting that E2F6 indeed inhibits the expression of CRNDE at the transcriptional level. Consequently, a series of functional experiments supported the hypothesis that CRNDE is downregulated by the transcription factor E2F6, which increases autophagy and inhibits apoptosis of GC cells. Nevertheless, the underlying downstream regulatory mechanism of CRNDE requires further exploration.

## Conclusion

CRNDE was downregulated in chemoresistant GC cells, thereby inducing autophagy and chemoresistance in GC cells. E2F6 transcriptionally repressed the expression of CRNDE and participated in CRNDE-mediated chemotherapy sensitization by regulating autophagy. This investigation suggests that CRNDE represents a potential prognostic biomarker and a therapeutic target for gastric cancer.

## Supplementary Material

Supplementary figures.
