# Supplementary material for: The lncRNA CRNDE is regulated by E2F6 and sensitizes gastric cancer cells to chemotherapy by inhibiting autophagy
Source: J Cancer. 2022 Aug 8;13(10):3061–72. doi: 10.7150/jca.65871 (PMC9414014; doi:10.7150/jca.65871)
Supplement: Supplementary file 1 — Supplementary figures. [file jcav13p3061s1.pdf]

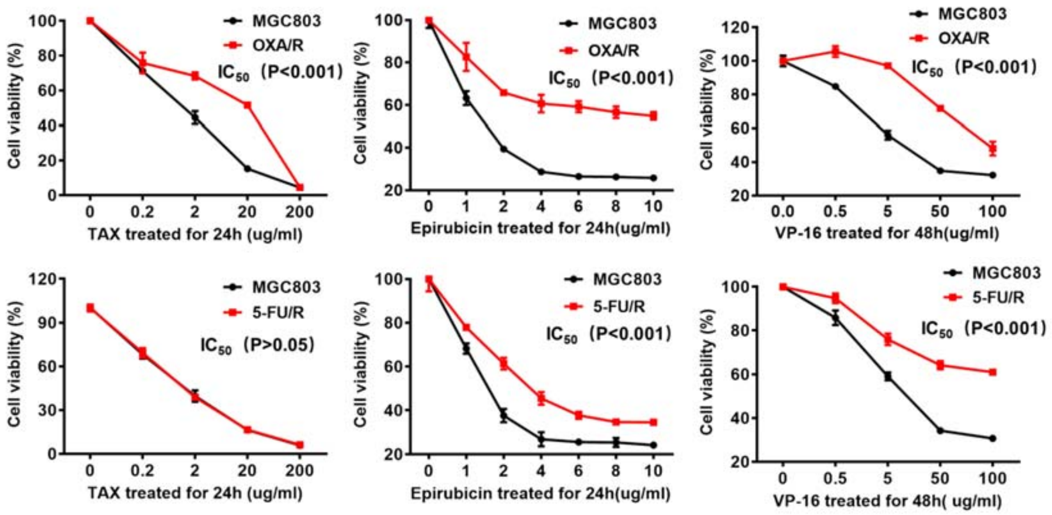

Supplementary figure 1. CCK8 drug sensitivity experiment was used to detect the cross-resistant of OXA/R and 5-FU/R to other chemotherapy drugs.

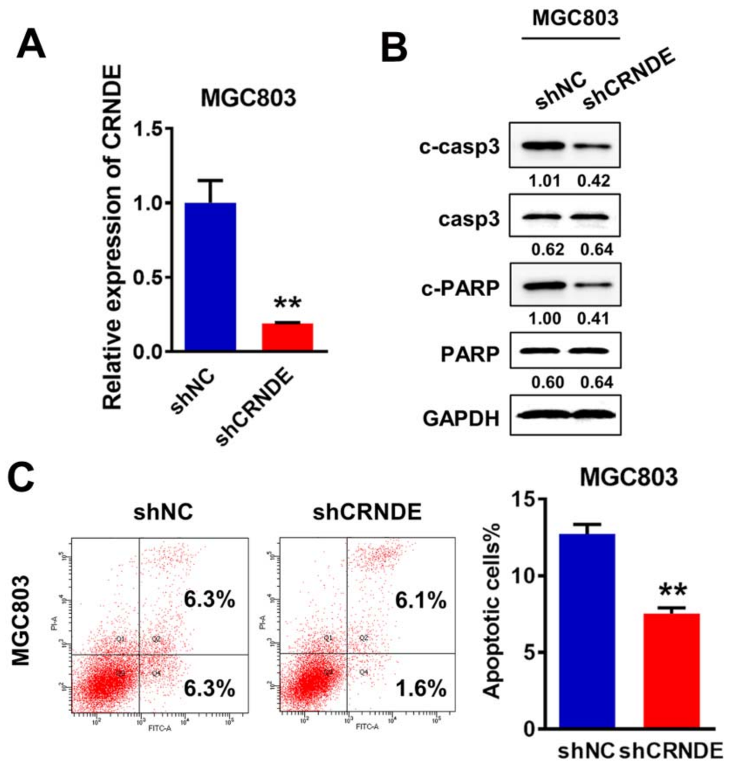

Supplementary figure 2. CRNDE silencing inhibited apoptosis of GC cells *in vitro*.

(A) RT-PCR analysis of CRNDE expression in MGC803 cells transfected with shCRNDE. (B) The protein levels of cleaved-caspase3 and cleaved-PARP in MGC803 cells transfected with shCRNDE were determined by western blot assay. (C) Flow cytometric analysis of apoptosis in MGC803 cells transfected with shCRNDE.
